# Efficacy of New Mindfulness-Based Swinging Technique Intervention: A Pilot Randomised Controlled Trial Among Women With Breast Cancer

**DOI:** 10.3389/fpsyg.2022.863857

**Published:** 2022-07-04

**Authors:** Ozan Bahcivan, Tania Estapé, Jose Gutierrez-Maldonado

**Affiliations:** ^1^Department of Clinical Psychology and Psychobiology, Faculty of Psychology, University of Barcelona, Barcelona, Spain; ^2^Psiko-Onkologlar Dernegi (Turkish Psycho-Oncological Association), Izmir, Turkey; ^3^FEFOC Foundation, Barcelona, Spain

**Keywords:** mindfulness, MBST®, breast cancer, self-efficacy, adherence to treatment, anxiety

## Abstract

**Objective:**

Combining 3rd-wave-therapies with Cognitive-Behavioural-Therapy (CBT) has increased in recent years. Usually these therapies require longer sessions which therefore increases the psychotherapy drop-out rate for cancer patients for multiple medical reasons. This inspired intervention of a shorter 20 min-long mindfulness-therapy (MBST) to be developed for Breast-Cancer-patients (BC).

**Method:**

This pilot randomised controlled trial was to assess the immediate-outcome of the MBST-intervention for its efficacy for BC-patients by using the Pearson Chi-square test, Fisher–Freeman–Halton exact test, and McNemar test for categorical variables; Mann–Whitney U and Wilcoxon test for the continuous variables. The Emotion Thermometer, State Trait Anxiety Inventory, Hospital Anxiety and Depression Scale, Self-Efficacy for Managing Chronic Disease, and Beck’s Hopelessness Scale were used for measuring the intervention outcomes. One hundred seventy-three BC patients were randomly assigned in two-groups (equal-mean-age, *p* = 0.417). Control-Group (CG, *n* = 82) received cognitive-disputation-technique a form-of-CBT, and Intervention-Group (IG, *n* = 74) received MBST. The directives are given to IG: psychoeducation about Mindfulness, and to imagine themselves swinging-in a peaceful environment. When the patients imagine their swing going up, they physically take a deep-breath, and when going down they physically release their breath, and this process is repeated.

**Result:**

Outcomes post-treatment showed significant higher-improvement in IG in all the assessed-measurements, with large-effect-size: anxiety (*p* < 0,05, *r* = 0,67) and depression-levels (*p* < 0,05, *r* = 0,71); anxiety-trait (*p* < 0,05; *r* = 0,79) reduced, it increases self-efficacy for managing-disease (*p* < 0,05, *r* = 0,82) as-well-as hopefulness (*p* < 0,05, *r* = 0,61) and saturation-level measured by pulse-meter/oximeter (*p* < 0,05, *r* = 0,51).

**Conclusion:**

MBST is an efficacious intervention to reduce psychotherapy session time for immediate relief from clinical anxiety and hopelessness as well as increase self-efficacy and improve tranquillity for BC-women. It may have a particular clinical significance for supporting patient’s adherence to treatment. Although in this pilot sample MBST was found to be effective for short-term-outcome, its efficacy for longer-term-outcome should be examined in future trials. Additionally, breathing laps can be increased possibly for a greater result on rise of saturation levels of patients.

## Introduction

The most prevalent type of cancer among women in the world is breast cancer (Bray et al., 2018; Siegel et al., 2019), and it is no different in Turkey (Kilickap et al., 2017; World Health Organization, 2020). Huang and Shi (2016) emphasised the fact that nearly 60% of patients with breast cancer reported high levels of anxiety and illness related stress (Youlden et al., 2014), yet 25.6%–58% of the patients reported living with depression (Mitchell et al., 2011; Turon et al., 2019). Further, Duggleby et al. (2014) reported that lack of hope has been conversely associated with psychological distress, and anxiety among cancer patients (Revier et al., 2012). This may be because of the poor treatment adherence and a decrease in quality of life (QOL) that might lead to greater emotional distress (Schandl et al., 2018; Baziliansky and Cohen, 2020; Riis et al., 2020). Therefore, delivering a psycho-oncological technique, which aims to decrease the effect of cancer patients’ emotional wellbeing, such as hopefulness and anxiety (Wolanin, 2021) are highly crucial fundamental aspect of integrated and holistic breast cancer care (Jacobsen and Wagner, 2012).

There are several interventions that support women with breast cancer to be able to manage physical and psychological negative effects throughout the diagnosis and treatment process (Fisher et al., 2019; Salsman et al., 2019; Wellisch, 2021). It has been stated by a number of studies that most cancer patients are interested in trying different adjunct techniques for several reasons, such as reducing stress, improving the immune system, or better spiritual upbringing (Rehse and Pukrop, 2003; Bahcivan and Moss, 2018). A growing body of evidence in the current literature has demonstrated that psycho-oncological interventions play an efficient role for cancer patients in enhancing their coping skill (Richardson et al., 2017), self-efficacy skills (Merluzzi et al., 2019) and decreasing stress and emotional discomfort (Wellisch, 2021). Self-efficacy is defined as the confidence needed to be able to accomplish difficult tasks or to handle challenging situations (Bandura, 2013). Additionally, the reduced hope was positively related to mood (Rottmann et al., 2010) psychological adaptation (Pourhosein and Farsham, 2021), emotional (Grealish et al., 2019), physical (Hoffman et al., 2009), and social wellbeing (Grealish et al., 2019) among a mixed group of people with cancer.

According to Kapogiannis et al. (2018), and Palesh et al. (2018), psychosocial treatment could be an option among cancer patients for tackling the side effects of cancer treatment. A large body of evidence has shown the success of Cognitive Behavioural Therapy (CBT); hence, it is a highly suggested primary choice of psychological therapy approach in treating depression [National Institute for Health and Clinical Excellence (NICE), 2009; Vanzeler, 2020]. Nonetheless, the CBT shows greater effectiveness when it is used in long term treatments (Arch et al., 2012; Hayes and Hofmann, 2017). Therefore, Sperry and Binensztok (2019) strongly recommended the adaptation of ultra-brief interventions, such as cognitive disputation (CD) technique which can be delivered in 10–20 min. This is particularly applicable to patients who suffer from a chronic illness. The CD technique which is a form of CBT (CBT-CD) aims to support patients to understand their own thoughts and emotions as just assumptions, but not interpret them as facts, this therefore potentially results in decreased anxiety and increased coping (Sperry and Sperry, 2017). This may be one of the reasons why many practitioners use CBT integrated with other evidence-based interventions, such as “third wave/generation” therapies, particularly for cancer patients (Hunot et al., 2010; Aksan, 2021).

One of the well-known third wave approaches is considered as Mindfulness-Based Cognitive Therapy (MBCT; Garland et al., 2014; Querstret et al., 2020), and Mindfulness-Based Stress Reduction (MBSR), which has been proven to have a reducing effect on distress as well as increasing psychological health for both non-cancer (Querstret et al., 2020), and cancer patients (Tang et al., 2015) including favourable changes to cerebral blood flow which results in reduced anxiety (Monti et al., 2012). Additionally, Mindfulness-Based Swinging Technique (MBST; Bahcivan et al., 2018) intervention combines a particular guided imagery for swinging activity inspired by *t’ai chi* and *qigong* motions. A systematic review conducted by Zimmermann et al. (2018), stated that several mindfulness interventions showed high acceptance rate for their effectiveness in regard to easing cancer patients’ anxiety and depression symptoms. Mindfulness is described as being aware of the moment with no prejudice, in fact it can be seen as being an ability that can be learned by practising (Crane et al., 2020).

Implementation of guided imagery (GI) with mindfulness technique is considered as an inseparable practice (Walker et al., 1999) that increases QOL (Charalambous et al., 2015). Visualisation is a complementary technique that is widely used (Walker et al., 1999; Gawain, 2016). This technique is easy, suitable, and not restraint of cancer patients’ activity levels (Chen et al., 2015). Research in the current cancer literature has proven that GI techniques enable breast cancer patients to relax, which has been shown to be beneficial in alleviating the adverse side effects of chemotherapy, such as sickness, vomiting (Samami et al., 2022), anxiety and stress (Mahdizadeh et al., 2019).

On the other hand, while transferring such skills to breast cancer patients; it is important to integrate imagery that promotes hope as it has a positive effect on patient’s stress level. Reduced hope may result in decreased self-efficacy (Duggleby et al., 2014). In spite of the fact that self-efficacy is not a fundamental part of mindfulness theory, it might still be good to take it into account as a possible mechanism (Bogosian et al., 2016). Mindfulness gives individuals the confidence in order to promote a ‘non-striving’ attitude, which can produce better decisions (Bogosian et al., 2016).

Previous randomised (Compen et al., 2019; AhmadiQaragezlou et al., 2020) and non-randomised (Cheli et al., 2020; Elimimian et al., 2020) mindfulness studies have demonstrated that cancer patients who received medical treatment displayed less symptoms of stress and psychological distress after mindfulness interventions (Branstrom et al., 2012). Heart Rate (HR) in beats-per-minute (bpm) can be seen as a bio-indicator of psychological distress and anxiety (Thayer et al., 2012), that should be monitored regularly even by mental health practitioners. During the mindfulness practice, imagining themselves in a peaceful environment may play a role in the change of HR (bpm; Lorca et al., 2019). Additionally, Xue et al. (2020) stated that, such interventions play role in increasing saturation level (SpO_2_) of individuals. In fact, RCT studies conducted by Beng et al. (2019) and Ng et al. (2016) used psychophysiological indicators, such as saturation level as an indicator for cancer patients’ perceived stress and anxiety symptoms which were significantly lessened after a short duration of breathing-exercise based mindfulness intervention.

One of the common reported findings were that; the mindful practice results in weakening the amygdala in regard to its response for emotional (Lutz et al., 2014) and resting state (Desbordes et al., 2012), meaning a calmer emotional stimulation (Tang et al., 2015). Similarly, preliminary evidence in the current field suggests that prefrontal cortex can be activated by meditation, and it could also arouse enhanced HR (bpm; Nolan et al., 2005; Xue et al., 2020). Intervention that used deep breathing is efficient in decreasing the HR (bpm) among essential hypertension patients (Kaushik et al., 2006) and provides relief from chemotherapy induced nausea (Aybar et al., 2020). Several research in the current field that had the intension to ease the symptoms experienced by cancer patients utilised relaxation techniques including qigong (Oh et al., 2012) and Progressive Muscle Relaxation (PMR; Demiralp et al., 2010).

Medical Qigong (MQ) comprises movement, breathing and meditation (Will, 2013). MQ is practiced with various activities, such as t’ai chi in the area of supportive oncology research and practice (Jahnke et al., 2010; Oh et al., 2012). In fact, MQ practices has the capacity to decrease depression, anxiety, and complaints of fatigue (Sagaonkar and Pattanshetty, 2021). Due to the qigong practice, patients’ who receive chemotherapy have shown development of better cognitive functioning (Oh et al., 2012). Will (2013) reported that MQ can be practiced by cancer patients during their treatment. This shows that, a repetitive movement of swinging motion has been linked with relaxation and calming (Osypiuk et al., 2020).

There were consistent findings from other randomised studies showing that there was an association between practicing mindfulness for 6 weeks (Lengacher et al., 2009; El-Deeb et al., 2016) or 8 weeks (Carlson and Garland, 2005) and improvement in the symptoms of depression and anxiety. However, Tang et al. (2015) suggested that future research should focus on the length of the mindfulness interventions; the RCT study assessing the immediate efficacy of mindfulness practices are needed in the psycho-oncology literature. In fact, Teo et al. (2019) recommended that shorter psychological interventions are more likely to assist patient’s adherence to their medical treatment.

The primary purpose of our study was to investigate the efficacy of this short brand-new mindfulness intervention called the Mindfulness Based Swinging Technique (MBST; Bahcivan et al., 2018). The MBST intervention combines a breathing exercise and a particular guided imagery for swinging activity inspired by *t’ai chi* and *qigong* motions that could make it possible to support women with breast cancer; for combating their anxiety, stress as well as increase their self-efficacy and hope. It is hypothesised that; this intervention which is as-short-as 20-min will increase patients’ perceived self-efficacy and their hope about their treatment and alleviate anxiety as well as increase patient’s oxygen saturation (SpO_2_) level and decrease heart rate (bpm).

## Materials and Methods

### Design

This is a pilot randomised controlled trial to test the immediate efficacy of Mindfulness Based Swinging Technique (MBST). The term “immediate” refers to no follow-up analysis has been done, days or weeks after the MBST intervention, the only follow up has been done only immediately after the MBST. This trial registered at the U.S. National Library of Medicine Registry, ClinicalTrials.gov *identifier* NCT03985267. This pilot randomised controlled trial was conducted by closely following the *CONSORT* (Consolidated Standards of Reporting Trials) *2010 guidelines statement extension to randomised pilot and feasibility trials* (Eldridge et al., 2016) respectively. All patients included in this pilot study have signed an informed consent.

### Participants

The study sample consisted of 173 women who met the following inclusion criteria: (a) women diagnosed with breast cancer, (b) who can consent, (c) native Turkish speakers, (d) currently under cancer treatment, (e) score at least 16 points for Hospital Anxiety and Depression scale (8 for anxiety, 8 for depression), (f) score maximum 7 points for Self-Efficacy for Managing Chronic Disease (in overall), (g) score at least 4 points for the Beck’s Hopelessness Scale (in overall), (h) score at least 40 points for State Trait Anxiety Inventory.

**Figure 1:**
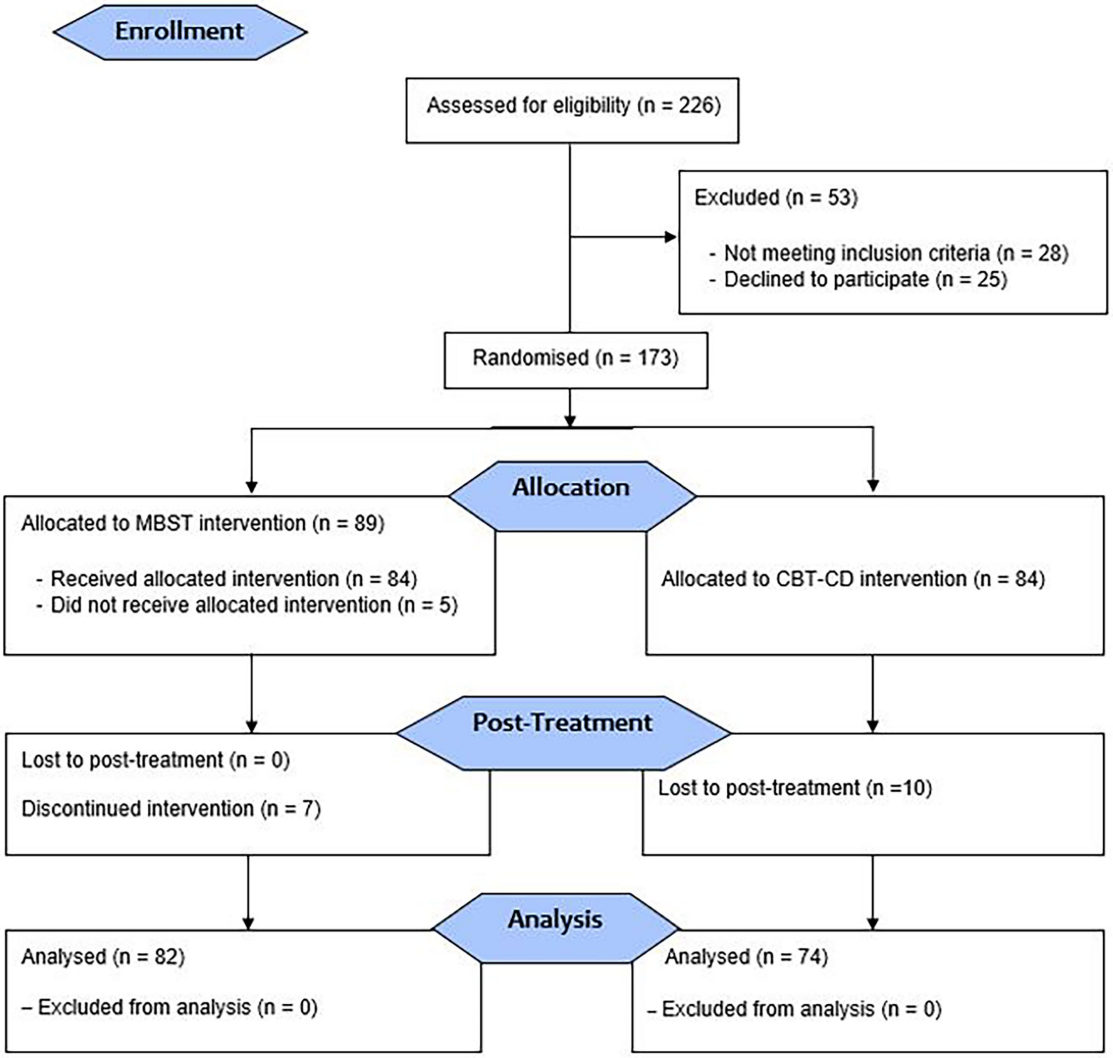
Flow diagram showing the participants’ selection, allocation and post-treatment.

Eighty-four participants were allocated into a control group, and 89 participants allocated into an experimental group. There were seven participants who discontinued the intervention from the experimental group. The discontinuation of the intervention means participants who completed the pre-tests but did not complete the MBST intervention. Yet, 10 participants from the control group lost-to-post-treatment. Lost-to-post-treatment means, participants who completed pre-tests and participated in the CBT-CD intervention but did not complete the post-tests (Figure 1). These total of 17 participants were not included in the analysis, and an additional drop-out analysis was not performed. In fact, participants who were included in the analyses completed all the required questionnaires. The participants were enrolled from March 2019 to August 2021, the intervention and the post-treatment periods began at the same time of the enrolment of the study.

### Intervention

The Mindfulness-Based Swinging Technique (MBST) intervention was administered to the eligible participants right after the self-administered psycho-social assessment for 20 min by the instructor. The MBST intervention session included a breathing exercise and a particular guided imagery for swinging activity as previously described (Bahcivan et al., 2018). Additionally, a 5-min brief psychoeducation about the nature of mindfulness, and instructions for the intervention were provided before the MBST intervention began by the instructor. Participants in the control group have taken the same self-administered psycho-social assessment but did not take part in the MBST, instead they received 20 min of CBT-CD which was formerly described (Horne and Watson, 2011; Sperry and Binensztok, 2019). Further treatment protocol about CD is explained by Sperry and Sperry (2017). There was no time interval for participants’ allocation and taking part in the intervention as it happened in the same day.

### Intervention Instructor

The Mindfulness Based *Swinging Effect* Technique (MBST) intervention instructed and applied by a culturally competent researcher who is a professionally qualified and experienced *Health Psychologist* with an additional training in “*Mindfulness in Therapeutic Practices”* and *“Mindfulness Based Cognitive Behavioural Therapy*.”

### Outcome Assessment

Participants were first randomised and then completed the psycho-social instruments about distress, anxiety, depression symptoms, self-efficacy, and hopefulness, as well as received measurements of heart rate (bpm) and oxygen saturation (SpO_2_) level right before commencing the intervention, and right after completing the intervention. The questionnaires were self-administered at the hospital and psychological consultancy centre on both occasions.

### Primary Outcome

#### Distress and Anxiety Symptoms

The Emotion Thermometer (ET) and State Trait Anxiety Inventory (STAI) were used for assessing the distress and anxiety symptoms. The ET developed by Mitchell et al. (2010) which consists of 5-visual analogue scales that measure four emotional domains including distress, anxiety, depression, anger and one outcome domain which is “need for help” utilised among cancer population. Each of the four emotional area scales is rated from 0 (none) to 10 (extreme). Mitchell et al. (2010) found an optimal balance between sensitivity and specificity. Participants were requested to pick the number that best reflects their level of emotion.

The Turkish adaptation of ET was done by Bahcivan and Eyrenci (2018). Their adaptation reported the overall Cronbach’s alpha of 0.87. The optimal cut-off score of 4 for depression thermometer of ET, and 5 for both anxiety and distress thermometers of ET yielded the optimal sensitivity and specificity values (sensitivity scores: 0.86, 0.75, 0.73 and specificity scores: 0.70, 0.68, 0.67 respectively). They concluded that the scale is an acceptable and practical tool for psychological distress screening among cancer patients.

The STAI consisted of two 20-item subscales measures state anxiety and trait anxiety (Spielberger et al., 1983). The STAI is self-administered on a four-point scale for each item, the patients were asked to indicate about how they feel for each of the 40 items. Each of the subscale score ranged from minimum 20 to maximum 80, the higher scores indicate the greater psychological anxiety. The internal consistency was 0.95. The Turkish adaptation of the STAI was done by Oner and Le Compte (1983). It is found to be a valid and reliable psychometric tool to use among patients. They indicated the internal reliability as 0.72, and test-re-test reliability as 0.86.

### Secondary Outcomes

#### Depression Symptoms

Hospital Anxiety and Depression Scale (HADS) was used for assessing depression and anxiety symptoms. HADS was developed by Zigmond and Snaith (1983) consists of 14-questions and is used to measure detecting states of depression and anxiety in clinical settings. The validity and reliability of the Turkish-language version were established by Aydemir et al. (1997). The items are scored on a Likert scale ranging from 0 (strongly disagree) to 3 (strongly agree). Measuring cut-off points of 8 for both anxiety and depression were used, respectively, to adapt the cultural norms (Miljanović et al., 2017). Many oncology settings use HADS instrument for its validity and reliability reasons (Clover et al., 2020).

#### Self-Efficacy

Self-Efficacy for Managing Chronic Disease (SEMCD) was used for assessing self-efficacy specifically targeting the management of chronic diseases. The six-item version of the SEMCD was developed and validated by Lorig et al. (2001). SEMCD consists of 10 sub-dimensions on a 10-point Likert-type scale “1” being the “not at all confident” and “10” being the “totally confident.” The higher score indicates increase management in self-efficacy about their chronic disease. The 6-item version was adapted to the Turkish language and culture by Incirkuş and Nahcivan (2020). According to this study, they indicated the Cronbach alpha values for the reliability as 0.95 for the SEMCD-total score and reported that it is a reliable and valid tool for clinical practice among Turkish patients.

#### Hopefulness

Beck’s Hopelessness Scale (BHS) was used for assessing hopefulness. The initial scale developed by Beck et al. (1974), the internal consistency of their study found to be high with Cronbach’s alpha being 0.85, this shows its reliability. The adaptation of the scale to Turkish language was performed by Durak and Palabiyikoglu (1994). According to the studies done, Cronbach alpha internal consistency coefficient of scale was 0.85, two-half reliability coefficient was 0.85 and test–retest reliability was 0.74.

#### Sample Size

The sample size was obtained during the study planning (see Supplementary Material). The necessary subjects’ numbers were determined as minimum of 45 for each group to be able to reject the null hypothesis that the population means of the experimental and control groups are equal with probability (power) 0,9. The Type-I error probability associated with this test of the null hypothesis is 0,05.

#### Randomisation, Allocation, and Concealment

The participants who met the eligibility requirements and signed the informed consent were randomly assigned either to the experimental (MBST intervention) or the control group (CBT-CD intervention; Figure 1). The random allocation sequence and assigning as well as the eligible 81-participants from the EgeMed Hospital in Aydin, Turkey and 75-participants were recruited from Ozel Oz Psikoloji Aile Danisma Merkezi (Oz Psychology Family Counselling Centre) in Izmir, Turkey, then generated by authorised staff of the recruiting institution. The numbers represent the patients’ admission sequence. Randomisation was performed through computer-generated list of random numbers. The study outcomes were assessed by self-administered questionnaires after the randomisation with the support from the researcher psychologist.

### Statistical Analysis

The results of this study were presented first by the sample descriptive, using mean and standard deviation for quantitative analysis, and frequencies for qualitative data (Table 1). The CONSORT guidelines were followed for describing the study flow (Figure 1). The dependence between the categorical variables were tested with the Pearson Chi-square test when the prerequisites (the expected number of observations in each cell must be 5 or greater than) were met, and otherwise the Fisher–Freeman–Halton exact test was used. Comparison of pre- and post-categorical variables within the control and experimental groups were performed by McNemar test. Then, pre- and post-measurements were compared within each group (in the control and intervention groups themselves). McNemar-Bowker Test was conducted for categorical variables. Since the assumption of normality was violated for continuous variables, all were tested with Wilcoxon test. Scores of pre and post differences were then calculated (by subtracting the pre-measures from the post measures for each patient), and Mann–Whitney U was tested (or Kruskal–Wallis analysis of variance [ANOVA]) to see if there were any differences in the distribution of the differences. Spearman’s rank order correlation was calculated for main variables. We used SPSS version 24 software packages to its statistical analysis and *p* < 0.05 was considered statistically significant. For multiple comparisons, Bonferroni correction was used to control the type I error rate (the significance level was determined by simply dividing the original significance level by the number of tests which was performed). In addition, *r* and *epsilon*-squared (*ε*^2^) effect sizes were calculated for each interaction; Z values divided by squared root the number of observations and chi-square are divided by one minus the number of observations. Effect size of *r* = 0.10 - < 0.30 and *ε*^2^ = 0.01 - < 0.08 are considered small, *r* = 0.30 - < 0.50 and *ε*^2^ = 0.08 - < 0.26 are considered medium and *r* ≥ 0.50 and *ε*^2^ ≥ 0.26 are considered large (Cohen, 1988).

**Table 1:** Demographic data of the two study groups.

Variable	Control *n* (%)	Intervention (MBST) *n* (%)	*Total*	*p*
Age (years)	52.92 (SD 9.62)	51.39 (SD 9.15)	52.29 (SD = 9.18)	.417^a^
Marital status				
Single	28 (37.8%)	25 (30.5%)	53	.333^b^
Married	46 (62.2%)	57 (69.5%)	103	
Current city				
Izmir	28 (37.8%)	52 (63.4%)	80	<.001^b^
Aydin	31 (41.9%)	12 (14.6%)	43	
Manisa	15 (20.3%)	18 (21.9%)	33	
Treatment center				
Hospital	39 (52.7%)	42 (51.2%)	81	.853^b^
Psychological consultancy centre	35 (47.3%)	40 (48.8%)	75	
Living status				
Alone	12 (16.2%)	15 (18.3%)	27	.732^b^
W/someone	62 (83.8%)	67 (81.7%)	129	
Education level				
Elementary	13 (17.6%)	15 (18.3%)	28	.112^b^
High school	27 (36.5%)	42 (51.2%)	69	
Bachelor or higher	34 (45.9%)	25 (30.5%)	59	
Employment status				
Employed	38 (51.4%)	30 (36.6%)	68	0.063^b^
Unemployed	36 (48.6%)	52 (63.4%)	88	
Smoking habit				
Smoker	19 (25.7%)	23 (28.0%)	42	.739^b^
N/Smoker	55 (74.3%)	59 (72.0%)	114	
Learning diagnosis				
1 month<	2 (2.7%)	4 (4.9%)	6	.060^c^
1–3 months	14 (18.9%)	8 (9.8%)	22	
3–6 months	15 (20.3%)	15 (18.3%)	30	
6 months–1 year	20 (27.0%)	13 (15.9%)	33	
1 year>	23 (31.1%)	42 (51.2%)	65	
Metastasis				
Yes	41 (55.4%)	35 (42.7%)	76	.112^b^
No	33 (44.6%)	47 (57.3%)	80	

*SD, standard deviation and HR, heart rate*.

a *Mann-Whitney U test*.

b *χ*^2^ *test*.

c *Fisher-Freeman–Halton exact test*.

## Results

The descriptive characteristics of the breast cancer patients are summarised in Table 1. There were not statistically significant (*p* > 0.05) differences between the intervention and control groups in terms of all demographic variables except the current city.

Within the scope of the primary outcome of the pilot study, pre-and post-measurements scores (within groups), the pre-measurements scores (between groups), and the differences between pre and post measurement scores (between groups) were analysed. These results listed in Tables 2 and 3. The pre-measurements of the groups are similar for most, with significant differences in three variables: *anxiety* (only STAI scores) and *need help* (ET) of the control group is higher than the intervention group, vice versa for self-efficacy level. Pre- and post- measurements were compared in both groups. The method in the control group (CBT-CD intervention) has no significant effect on SpO_2_ level, HADD scores, and depression level (ET). On the other hand, there was significant difference between pre and post tests for all variables in the MBST intervention group. In addition, the post-test scores of the groups were compared, it was seen that, there were significant differences between control and intervention groups. A similar difference was also seen in the categories of the hope variable in favour of the experimental group (Table 2).

**Table 2:** Frequency for hope and comparing the control and intervention groups.

Variable	Control *n* (%)	Intervention (MBST) *n* (%)	Total	*p*
Hope (pre)				
Hopeful	8 (10.8%)	10 (12.2%)	18	0.421^a^
Hopeless	30 (40.5%)	25 (30.5%)	55	
Unsure	36 (48.6%)	47 (57.3%)	83	
Hope (post)				
Hopeful	21 (28.4%)	71 (86.6%)	92	<0.001^a^
Hopeless	16 (21.6%)	3 (3.7%)	19	
Unsure	37 (50.0%)	8 (9.8%)	45	
The comparison pre- and post-measurements’ value of *p*	<0.05^b^	<0.001^b^		

a *χ^2^ test*.

b *McNemar-Bowker Test*.

**Table 3:** Means (SD) and summary statistics (Mann–Whitney U and Wilcoxon test) for pilot study variables comparing control and intervention group across pre- and post-measures.

	Control Group				Intervention Group				Pre-measurement comparison		Post-measurement comparison		Gain scores comparison	
	Pre	Post	*Z*^†^	*r*	Pre	Post	*Z*^†^	*r*	*Z*^‡^	*r*	*Z*^‡^	*r*	*Z*^‡^	*r*
	M (SD)	M (SD)			M (SD)	M (SD)								
HR (bpm)	94.43 (3.29)	92.65 (4.03)	−4.78^*^	0.39	94.21 (3.76)	85.98 (3.46)	−7.87^*^	0.61	−0.11	0.01	−8.55^*^	0.68	−8.98^*^	0.72
Oxygen saturation (SpO_2_)	94.50 (2.97)	94.60 (3.16)	−0.51	0.04	95.15 (2.48)	97.11 (1.85)	−6.73^*^	0.53	−1.46	0.12	−5.10^*^	0.41	−6.42^*^	0.51
Distrees (ET)	6.30 (1.21)	5.26 (1.01)	−6.92^*^	0.57	6.28 (1.83)	2.72 (1.67)	−7.68^*^	0.60	−0.30	0.02	−8.56^*^	0.69	−8.19^*^	0.66
Anxiety														
HADA	12.15 (2.39)	11.39 (1.69)	−3.70^*^	0.30	12.23 (3.01)	7.15 (3.06)	−7.69^*^	0.60	0.00	0.00	−8.19^*^	0.66	−8.42^*^	0.67
STAI	48.94 (5.24)	45.01 (6.05)	−5.82^*^	0.48	43.13 (5.16)	25.73 (5.88)	−7.79^*^	0.61	−4.48^*^	0.36	−10.51^*^	0.84	−9.83^*^	0.79
Anxiety (ET)	6.28 (1.01)	5.51 (1.17)	−5.27^*^	0.43	6.50 (1.65)	3.01 (1.68)	−7.67^*^	0.60	−0.62	0.05	−8.40^*^	0.67	−8.60^*^	0.69
Depres														
HADD	12.00 (1.80)	11.55 (2.17)	−1.95	0.16	11.26 (2.20)	6.49 (2.49)	−7.69^*^	0.60	−1.80	0.14	−9.42^*^	0.75	−8.91^*^	0.71
Depres. (ET)	5.72 (0.97)	5.65 (1.05)	−0.57	0.05	5.79 (1.57)	3.51 (1.43)	−7.27^*^	0.57	−0.17	0.01	−9.05^*^	0.72	−8.48^*^	0.68
Self-efficacy	5.64 (0.77)	5.94 (0.79)	−5.93^*^	0.49	5.96 (0.89)	8.10 (1.27)	−7.87^*^	0.61	−2.32^*^	0.19	−8.95^*^	0.72	−10.22^*^	0.82
Hopelesness	10.08 (2.32)	8.81 (2.30)	−5.57^*^	0.46	10.51 (3.60)	6.02 (4.20)	−7.69^*^	0.60	−0.59	0.05	−5.23^*^	0.42	−7.60^*^	0.61
Anger (ET)	5.78 (1.17)	5.49 (1.41)	−2.45^*^	0.20	5.18 (2.81)	3.09 (1.96)	−6.70^*^	0.52	−1.25	0.10	−7.11^*^	0.57	−6.58^*^	0.53
Help (ET)	6.88 (1.36)	5.22 (1.67)	−5.52^*^	0.45	5.84 (2.58)	3.67 (2.09)	−7.39^*^	0.58	−2.60^*^	0.21	−4.95^*^	0.40	−2.06^*^	0.16

* *p < 0.05*.

† *Wilcoxon signed rank test*.

‡ *Mann–Whitney U test*.

Since pre-tests scores differed between control and intervention groups, the groups were compared with gain scores calculated by subtracting the pre-tests scores from the post-tests scores within each group to be compared (Hegde and Salvatore, 2021). As seen in Table 3, large, significant differences were observed for the intervention group on HR (bpm), SpO_2_ level, the distress level (ET), HADA, STAI, anxiety level (ET), HADD scores, depression level (ET), self-efficacy level, hopelessness level, and anger level. Only small, significant differences were observed on need help level (ET). According to these results, HR (bpm), anxiety, depression (for all depression measurements), hopelessness, anger and need help scores significantly decreased, SpO_2_ and self-efficacy levels significantly increased in the intervention group compared to control group.

In order to test the first of secondary outcomes, correlations coefficient among the main variables separately for breast cancer patients in control and intervention groups were calculated in Table 4. As it can be seen for the intervention group’s post measures, self-efficacy had moderate significantly negative correlations with two anxiety measures; HADA scores, STAI scores, hopelessness, and weak negative correlations with one of the anxiety measures; anxiety level (ET), and depression measurements; HADD scores and depression level (ET). In addition, moderate significantly positive correlations were found between self-efficacy and SpO_2_ levels. Moreover, the correlation values for the intervention group in the last measurements increased in absolute value from the pre-test. Once the last measurements were compared with the control groups, the correlation values obtained for the intervention group were greater in absolute value.

**Table 4:** Spearman’s rank order correlations of self-efficacy with hopelessness, anxiety, depression, and saturation level (SpO_2_) among cancer patients.

			1	2	3	4	5	6	7
Control group	1. Self efficacy	Pre	-						
		Post	-						
	2. HADA	Pre	0.29^*^	-					
		Post	0.05	-					
	3. STAI	Pre	−0.12	0.08	-				
		Post	−0.25^*^	0.01	-				
	4. Anxiety (ET)	Pre	0.13	0.33^**^	0.24^*^	-			
		Post	0.19	0.32^**^	0.09	-			
	5. Hopelesness	Pre	0.16	0.14	0.16	0.25^*^	-		
		Post	0.18	0.56^**^	−0.09	0.45^**^	-		
	6. HADD	Pre	0.27^*^	0.31^**^	0.28^*^	0.14	0.13	-	
		Post	0.05	0.39^**^	0.12	0.03	0.11	-	
	7. Depression (ET)	Pre	0.21	0.03	0.23	0.31^**^	0.17	0.17	-
		Post	0.20	0.11	0.25^*^	0.35^**^	0.09	0.15	-
	8. SpO_2_	Pre	−0.06	0.01	0.14	0.30^**^	0.09	−0.05	0.32^**^
		Post	0.06	0.03	0.08	0.27^*^	0.12	−0.07	0.33^**^
Intervention group	1. Self efficacy	Pre	-						
		Post	-						
	2. HADA	Pre	−0.39^**^	-					
		Post	−0.48^**^	-					
	3. STAI	Pre	−0.41^**^	0.23^*^	-				
		Post	−0.54^**^	0.31^**^	-				
	4. Anxiety (ET)	Pre	−0.29^**^	0.34^**^	0.03	-			
		Post	−0.36^**^	0.39^**^	−0.02	-			
	5. Hopelesness	Pre	−0.17	0.16	−0.14	0.24^*^	-		
		Post	−0.48^**^	0.71^**^	0.41^**^	0.45^**^	-		
	6. HADD	Pre	−0.26^*^	0.55^**^	0.27^*^	−0.04	0.1	-	
		Post	−0.30^**^	0.49^**^	0.17	0.22	0.43^**^	-	
	7. Depression (ET)	Pre	−0.25^*^	0.36^**^	0.04	0.40^**^	0.01	−0.1	-
		Post	−0.38^**^	0.50^**^	0.1	0.43^**^	0.22^*^	0.47^**^	-
	8. SpO_2_	Pre	0.26^*^	−0.08	−0.35^**^	−0.12	0.09	−0.26^*^	−0.05
		Post	0.45^**^	−0.30^**^	−0.17	−0.26^*^	−0.07	−0.24^*^	−0.42^**^

*Pre, pre-measurement; post, post-measurement; ET, emotion thermometer; HADA, Hospital Anxiety and Depression Scale (Anxiety); HADD, Hospital Anxiety and Depression Scale (Depression); STAI, State Trait Anxiety Inventory; and SpO_2_, Oxygen Saturation Level*.

* *p* *< 0.05;*

** *p < 0.01*.

In order to test the other secondary outcome; Kruskall–Wallis and Mann–Whitney-U test were used (see Tables 5 and 6). For the control group, *education level*, *marital status*, *treatment centre*, *living arrangement* and *metastatic status* of the breast cancer patients had no significant impact on depression, anxiety, self-efficacy, and hopefulness except those two measurements of anxiety: STAI scores and anxiety (ET) for education level, STAI scores for *marital status* and self-efficacy for *metastatic status*. On the other hand, significant differences were found between the *time for leaning their diagnosis* scores on one of the anxiety measurements; HADA scores and all depression measures.

**Table 5:** The impact of demographic variables “a” on anxiety and depression scores, self-efficacy, and hopefulness.

	Education level			Marital status			Living arrangement		
	*χ*^2^^†^	*ε* ^2^	*Post-hoc*	*Z*^‡^	*r*	*Post-hoc*	*Z*^‡^	*r*	*Post-hoc*
*Control group*									
Anxiety									
HADA	0.41	0.00	-	−0.13	0.01	-	−0.21	0.02	-
STAI	13.21^**^	0.09	Hs < Bd	−2.55^*^	0.20	S > M	−0.65	0.05	-
Anxiety (ET)	9.36^**^	0.06	Hs < Bd	−0.42	0.03	-	−0.06	0.00	-
Depression									
HADD	1.81	0.01	-	−0.09	0.01	-	−0.36	0.03	-
Depres. (ET)	3.73	0.02	-	−0.64	0.05	-	−0.37	0.03	-
Self efficacy	0.44	0.00	-	−0.77	0.06	-	−1.34	0.11	-
Hopelesness	3.17	0.02	-	−0.61	0.05	-	−1.15	0.09	-
*Intervention group*									
Anxiety									
HADA	0.82	0.01	-	−3.78^*^	0.30	S < M	−3.72^*^	0.30	A < WS
STAI	2.79	0.02	-	−0.97	0.08	-	−0.24	0.02	-
Anxiety (ET)	5.11	0.03	-	−3.73^*^	0.30	S < M	−3.14^*^	0.25	A < WS
Depression									
HADD	7.83^**^	0.05	Ps > Bd	−1.96^*^	0.16	S < M	−2.60^*^	0.21	A < WS
Depres. (ET)	23.31^**^	0.15	Ps < Hs,Bd	−1.49	0.12	S < M	−0.42	0.03	-
Self efficacy	5.34	0.03	-	−5.29^*^	0.42	S < M	−3.82^*^	0.31	A < WS
Hopelesness	7.64^**^	0.05	Ps > Hs,Bd	−1.06	0.08	-	−2.20^*^	0.18	A < WS

**ε*^2^ Effect size for Kruskall–Wallis H test; r Effect size for Mann–Whitney U test. PS, Primary School; HS, High School; BD, Bachelor’s degree; S, Single; M, Married; A, Alone; and WS, With Someone*.

* *p < 0.05*.

** *Bonferroni corrected value of *p* set at *p* < 0.017*.

† *Kruskall–Wallis H test*.

‡ *Mann–Whitney U test*.

**Table 6:** The impact of demographic variables “b” on anxiety and depression scores, self-efficacy, and hopefulness.

	Treatment Centre			Time for learning their diagnosis			Metastatic Status		
	Z^‡^	*r*	*Post-hoc*	χ^2^^†^	*ε* ^2^	*Post-hoc*	Z^‡^	*r*	*Post-hoc*
*Control group*									
Anxiety									
HADA	−0.38	0.03	-	20.28^**^	0.13	1-3 m < 3-6 m, 6 m-1y,1 y>	−0.63	0.05	-
STAI	−1.27	0.10	-	6.87	0.04	-	−0.36	0.03	-
Anxiety (ET)	−0.11	0.01	-	3.77	0.02	-	−0.10	0.01	-
Depression									
HADD	−0.39	0.03	-	10.18^**^	0.07	1-3 m < 1 y>	−1.02	0.08	-
Depres. (ET)	−1.48	0.12	-	9.83^**^	0.06	<1 m > 6 m-1y	−0.34	0.03	-
Self efficacy	−0.60	0.05	-	4.03	0.03	-	−3.03^*^	0.24	Y > N
Hopelesness	−1.08	0.09	-	8.63	0.06	-	−0.41	0.03	-
*Intervention group*									
Anxiety									
HADA	−0.45	0.04	-	5.14	0.03	-	−1.74	0.14	-
STAI	−0.32	0.03	-	4.10	0.03	-	−1.20	0.10	-
Anxiety (ET)	−0.34	0.03	-	13.71^**^	0.09	<1 m, 1–3 m > 3-6 m	−1.91	0.15	-
Depression									
HADD	−0.35	0.03	-	18.05^**^	0.12	1-3 m > 3-6 m, 6 m-1y; 6 m-1y < 1y>	−1.28	0.10	-
Depres. (ET)	−0.62	0.05	-	11.71^**^	0.08	<1 m < 6 m-1y	−1.38	0.11	-
Self efficacy	−1.07	0.09	-	23.94^**^	0.15	3-6 m < 1–3 m, 6 m-1y, 1y>	−3.30^*^	0.26	Y < N
Hopelesness	−0.22	0.02	-	12.37^**^	0.08	3-6 m < 1y>	−3.68^*^	0.29	Y < N

**ε*^2^ Effect size for Kruskall–Wallis H test; r Effect size for Mann–Whitney U test; <1 m, less 1 month; 1–3 m, 1–3 month; 3-6 m, 3–6 month; 6 m-1y, 6 month–1 year; 1y >, more than 1 year; Y, Yes; and N, No*.

* *p < 0.05*.

** *Bonferroni corrected value of *p* set at *p* < 0.005*.

† *Kruskall–Wallis H test*.

‡ *Mann–Whitney U test*.

In the intervention group; depression, anxiety, and self-efficacy scores differ significantly according to *education level*, *marital status*, *treatment centre*, *living arrangement* and *metastatic status* of the breast cancer patients, but there are some exceptions: all measurements of anxiety for *education level*, STAI scores, depression level (ET) for *marital status*, STAI scores and depression level (ET) for *living arrangement*, two measurements of anxiety; HADA scores and anxiety level (ET) for *metastatic status*. Furthermore, significant differences were found between *time for learning their diagnosis* for anxiety (except for HADA and STAI scores) and all the depression measurements. For hopelessness, education level, *time for learning their diagnosis* (X^2^(3) =17.47; *p* < 0.05; *ε*^2^ = 0.31), and *metastatic status* have significant impact, except for *marital status* and *living arrangement*.

### Acceptability of the Pilot Study

There are no significant differences between the centres (hospital or private clinic) where both MBST and CBT-CD were applied among breast cancer patients (*p* = 0.853). The MBST and CBT-CD can be applied to any breast cancer patients who are at the age of 18 and above (*p > 0*.05). The breast cancer patients who smoke also benefit from MBST (*p > 0*.05). The patients who learned about their cancer diagnosis within the 3-months of time showed greater results in efficaciousness for MBST than patients who learned about their diagnosis for more than 3-months. On the other hand, CBT-CD seems to be more efficacious for patients who learned their diagnosis for more than 3 months. Having any metastasis has no impact in conducting MBST nor CBT-CD for breast cancer patients (*p > 0*.05). In terms of acceptability rating for MBST intervention, the hope scores were increased by 74.4% (*p < 0*.001).

## Discussion

This is the first pilot randomised controlled trial examined the immediate efficacy of Mindfulness Based Swinging Technique (MBST). The current findings suggest that 20 min long MBST intervention may have immediate efficacy for women with breast cancer. The “immediate” means there were no follow-up analysis has been done, days or weeks after the intervention, only immediately after it. The participants who received MBST reported significantly reduced perceived stress, anxiety and depression scores, and increased hopefulness and perceived self-efficacy, which has similar outcomes with the earlier randomised (Kenne-Sarenmalm et al., 2017; Compen et al., 2019; Lorca et al., 2019; Shao et al., 2020) and non-randomised (Witek-Janusek et al., 2008; Monti et al., 2012) mindfulness studies, except MBST has a greater reduced length in intervention time.

Lemanne and Maizes (2018) claim that there are mixed findings regarding the usefulness of guided imagery for alleviating stress, anxiety, and depression in cancer patients (Redd et al., 2001). The measurement tools which were utilised in this research have similarities with other psycho-oncological research (Montazeri, 2008; Kenne-Sarenmalm et al., 2017) as these studies also employed HAD for measuring anxiety and depression scores among cancer patients. Gawain (2016) argue, there are still gaps in the literature in respect of the efficacy of relaxation and imagery techniques for comforting anxiety and depression symptoms among cancer patients. Fortunately, these techniques have been evaluated in the current pilot study, as we aimed to contribute to the efficacy of guided-imagery intervention among breast cancer patients. For this matter, the guided-imagery has been an integrated part of the MBST intervention (Bahcivan et al., 2018), which showed immediate efficacy for the abovementioned areas of psychological discomfort.

The CBT-CD was found efficacious in various levels between low to middle in Heart Rate (HR), anxiety, and hopelessness, whereas; the MBST had shown much higher immediate efficacy in these domains. Thus far, the CBT-CD has no immediate efficacy on depression symptoms, anger, and SpO_2_ level, whilst MBST had shown better immediate efficacy. Yet, it should be noted that, there is a greater chance that patients’ attitude toward the intervention could have been an influence in order to make improvements in their overall mental health (Ledesma and Kumano, 2009; Lederberg et al., 2015). Nevertheless, pre-post comparison tests of the MBST results showed immediate efficacy in all domains including hopelessness, SpO_2_ level and HR (bpm). In fact, our results indicated that conducting the MBST in different centres had the similar efficaciousness.

Further, being hopeful can be explained as a psychosocial domain that quite possibly exists when the level of self-efficacy is high, and sorrow is low. This can be interpreted as hopefulness may influence physical and mental health in a positive way (Kabat-Zinn, 2011; Duggleby et al., 2014). The studies conducted by Duggleby et al. (2014); Merluzzi et al. (2019) favour our findings, as their results indicated that there was a positive correlation between patients’ self-efficacy, level of hopefulness and being diagnosed with breast cancer (Duggleby et al., 2014; Merluzzi et al., 2019) therefore, these correlations could be seen as predictors in patient’s general wellbeing for mental health professionals.

Unsimilar to CBT-CD intervention, the MBST intervention can be used at any time regardless of when the patients learn about their cancer diagnosis in order to reduce scores for their immediate depression and anxiety symptoms. However, in this pilot study, it has shown that CBT-CD seems to be superior, particularly for increasing self-efficacy and hopefulness of breast cancer patients regardless of when the patients learned about their cancer diagnosis. On the other hand, Rottmann et al. (2010) argued that education level can be considered as a predictor of socioeconomic status of patients. Meaning that, patients who have received better education showed higher self-efficacy and physical functioning (Rottmann et al., 2010). Furthermore, their results indicated patients who had a better education level perceive themselves more resilient in stressful situations and have better coping skills with stress compared to patients who received lower education. This supports Peuckmann et al. (2007)‘s findings which discovered that among Danish breast cancer survivors, low quality of life and shorter education were associated, respectively. In fact, Schandl et al. (2018) concluded that, particularly female cancer patients who have received lower education may be more vulnerable in regard to have a better quality of life. However, in our pilot study the MBST found to be an efficacious intervention immediately in lessening the depression scores regardless of education level of the patients, yet CBT-CD only found to be an efficacious intervention for easing the anxiety symptoms for all educational backgrounds. In fact, it should be noted that, according to anxiety scores obtained from STAI measurement, both CBT-CD and MBST interventions support alleviation in trait and state anxiety. However when compared to each other, MBST has proven itself to be superior to CBT-CD considering the state and trait anxiety of the patients within 20 min of time. Grepmair et al. (2007) argued that therapist’s general clinical and mindfulness-based experiences should be considered separately. This proposes that; the experience level of the therapist may have a direct or an indirect effect on participating patients’ clinical outcome (Grepmair et al., 2007; Norcross et al., 2013). Khoury et al. (2013) reported that many studies lack in providing information about the treatment moderator’s professional background or the number of treatment moderators that were included in their study. However, in our pilot study the treatment moderator’s professional and educational background, as well as mindfulness-based training transparently specified for better indication to the readers.

In terms of the physical measures, CBT-CD showed only for little immediate improvement for HR (bpm) and displayed no efficacy for SpO_2_ level. This was explained by Ledesma and Kumano (2009), as one of the elements that could have caused a small mean effect size may be due to determining the physical measurements after a very short period of time from the post intervention. Some mindfulness studies included physical health measurements in their research (Monti et al., 2006). Ledesma and Kumano (2009) stated that, there would be only a very small enhancement in the physical component, this might be due to the patients who are actively undergoing chemotherapy, radiotherapy, or different types of cancer treatment. Nevertheless, the MBST showed immediate better outcome for HR (bpm) and SpO_2_ level amongst breast cancer patients.

The present pilot study offers several theoretical, practical, and clinical contributions to the emerging field of psycho-oncology practice. For example, women with breast cancer who are in active cancer treatment can benefit from MBST as their stress, and anxiety symptoms may be immediately alleviated in some levels. This is particularly significant, because psychological interventions that targets oncology patients usually requires longer and multiple sessions to see a noticeable results. Moreover, our research supports guided imagery technique which was inspired by *t’ai chi* and *qigong* motions as an efficacious method to be used in psycho-oncological practice which plays a role in closing the gap in the current literature. Additionally, the MBST not only supports psychological, but also aids physical wellbeing by increasing the SpO_2_ level and taken part in regulating the HR (bpm) for women with breast cancer.

### Limitations, Strengths, and Future Research Implications

This study has some limitations. First, patients’ attitude toward a mindfulness intervention was not tested, and patients were still randomised regardless. Second, the therapist had multiple roles, such as implemented the intervention, administered the questionnaires, and analysed the data. On the other hand, the dropout rate was low in both the experimental and control groups, but lower in the experimental group as expected, since the MBST treatment was in a single session and lasted only for 20 min. This indicates the strength of the MBST intervention and shows its acceptability.

Since our results showed some promising immediate efficacy for one-on-one MBST among breast cancer patients, further research evaluating the MBST’s long-term efficacy should be conducted for both in group and one–one–one sessions. In fact, during the COVID-19 pandemic, it will be particularly significant to increase online psycho-oncological techniques; therefore the MBST should be evaluated for its efficacy as an e-health intervention in further research.

## Data Availability Statement

The datasets presented in this article are not readily available because of the ethical reasons the research data are not shared. Requests to access the datasets should be directed to OB, psikoonkoloji@yahoo.com.

## Ethics Statement

The studies involving human participants were reviewed and approved by the Research Ethics Committee of Nigde University (Decision Number: 2018/14–01). The patients/participants provided their written informed consent to participate in this study.

## Author Contributions

OB has made a substantial, direct, and intellectual contribution, TE and JG-M have equally supervised the entire work and approved it for publication.

## Funding

The authors declare that this study received funding from Oz Bireysel Danismanlik LTD STI to cover the publication cost. The funder was not involved in the study design, collection, analysis, interpretation of data, the writing of this article or the decision to submit it for publication.

## Conflict of Interest

The authors declare that the research was conducted in the absence of any commercial or financial relationships that could be construed as a potential conflict of interest.

## Publisher’s Note

All claims expressed in this article are solely those of the authors and do not necessarily represent those of their affiliated organizations, or those of the publisher, the editors and the reviewers. Any product that may be evaluated in this article, or claim that may be made by its manufacturer, is not guaranteed or endorsed by the publisher.
